# Vegetable Morphology

**Published:** 1837-10-01

**Authors:** 


### ON VEGETABLE MORPHOLOGY.

I. An Introduction to Botany. By *John Lindley*, F.R.S. L.S. & G.S., &c. Second edition. London, 1835.

II. Nouveau Systeme de Physiologie Vegetale et de Botanique. Par *F. V. Raspail*. 2 Tomes. Paris, 1837. Bailliere.

In the last number of this Journal, we attempted to explain some of the most interesting and instructive laws of Philosophical Anatomy, and to point out and illustrate that wonderful harmony of developement, which exists not only in the different parts and organs of the same animal body, but also in these parts in the different classes of animals, from the more simple up to the most complex of the scale. We then endeavoured to shew that most of the anomalies or congenital defects of formation are truly and strictly mere *arrests* of the process of evolution; that the part or organ so affected had been interrupted and stopped, as it were, in its progress to maturity; and that thus it exhibited that appearance or structure which is normal to the fœtus at a certain stage of its intra-uterine existence, but which is abnormal, or anomalous to it, after birth. Guided by the celebrated law of Centripetal Developement, we found ourselves enabled to account most satisfactorily for very many of these malformations, the nature and origin of which had hitherto been quite inexplicable. We then proceeded to illustrate the striking analogies which may be traced between the embryotic and early fœtal organization of the higher animals, and the mature or normal developement in animals, lower in the animal scale; and having, we hope, established the truth of these analogies, we were prepared to trace the resemblances between the congenital malformations of the human fœtus and the natural organization of many of the lower animals.

The last section of our inquiry was devoted to the investigation of the curious, and hitherto ill-understood subject of Hermaphrodism. We found that most of the bizarre irregularities of the generative organs, included under this appellation, are strictly obedient to the laws above mentioned; that very many of them are beautifully illustrative of M. Serres' great discovery of centripetal formation; and that others find their exact prototypes, in the normal structures of animals lower in the scale of developement.

It has occurred to us that it might be interesting to the philosophical reader to have his attention directed to the corresponding departments of Vegetable Anatomy; and with this view we now propose to devote a few pages to what has been called Morphology, or that subject of botanical research, whose object is to trace the phenomena of the developement of different parts of plants, and to search out the laws which these phenomena seem to obey.

It has been supposed that the higher and more complicated forms of vegetables pass, during their growth, through successive stages or phases of developement, and that each of these stages represent, as it were, and may be compared to, the permanent and mature forms of those plants, whose organization is more simple. If we examine the structure of some of the simplest cryptogamous plants, as the tribe of fungi or mushrooms, we find that it consists entirely of an aggregation of vesicles or minute cells, there being no, or only very indistinct, traces of a fibrous or vascular texture. Now the same holds good of the minute anatomy of the seed, before it has begun to germinate. It is strictly and truly of a cellular structure ; and we find no obvious traces of vessels in it, until the germinating process has been fairly established.

Again, an analogy has been traced between the structure of the embryo plant, when it has just forced up its plumula above the surface of the ground, and the structure discoverable in mosses; and, lastly, at a more advanced period of growth, when the proper leaves of the plant are first formed, but as yet no attempt at the formation of flowers or seed has taken place, we discover a resemblance to the structure of the ferns—the highest in the family of cryptogamic plants. It is proper, however, to observe, that considerable obscurity still rests on some of these speculations; but certainly, even if we had not the direct researches of such accomplished botanists, as De Candolle, Agardh, and others, to warrant the above conclusions, a strong presumptive proof in their favour is afforded by the splendid discoveries in the corresponding department of animal anatomy.

After these brief prefatory remarks, we now approach the subject of vegetable Morphology; and in order to make our descriptions as easily intelligible as possible, we propose to trace very shortly the process of growth of a perennial plant, from the early germination of its seed to the production of its stem, branches, leaves, and flowers.

When a seed is placed in moist earth, at a temperature above the freezing point, it speedily begins to imbibe moisture, and then gradually softens and swells out in all directions. It absorbs oxygen, gives out a portion of carbonic acid, and the fecula or starch, hitherto its chief constituent, is by degrees converted into saccharine matter—the proper nutriment or food of the vegetable embryo at this period of evolution. The radicle and the plumule are gradually protruded; the former descending and taking root in the earth, and the latter rising up above the surface of the ground, and carrying along with it the two cotyledons, which, after certain changes, assume somewhat the appearance, and also perform the functions—the aëration and elaboration of the sap—of the future leaves. At this stage of vegetable growth, the structure of the young plant has been found, as already mentioned, to be almost entirely composed of cellular tissue, the fibrous and vascular tissues being as yet but very imperfectly formed.

The plumule or nascent stem rises higher and higher—its growing point having already " acquired the rudimentary state of a leaf, which continues to develope as the plumule elongates, until, when the first internode* of the latter ceases to lengthen, the leaf has actually arrived at its complete formation."

The leaf, when expanded, performs the part of lungs to the growing vegetable; the sap, as it circulates through it, is exposed to the action of the air, and is thus prepared to form woody matter, which is then gradually

* The point of a stem or of a branch, from which the leaves arise, is called a *node;* and the space between any two of these points is called an *internode.*

deposited between the bark, and the medullary sheath or the membrane investing the pith.

Other leaves are successively formed at the point of the stem, as it grows upward; and this process continues to go on, until at length the stem ceases to lengthen. Then we discover that a new change takes place in the vegetable economy.

"The old leaves gradually fall off; the new leaves, instead of expanding after their formation, retain their rudimentary condition, harden, and fold over one another, so as to be a protection to the delicate point of growth; or, in other words, become the scales of a bud. We have now a shoot with a woody axis, and a distinct pith and bark, and of a more or less conical figure. At the axil of every leaf a bud had been generated during the growth of the axis; so that the shoot, when deprived of its leaves, is covered from end to end with little, symmetrically arranged, projecting points, which are the buds." 237.

On the return of the genial weather of Spring, the buds, which had been formed during the previous year, begin to expand and unfold, in consequence of the movement upwards of the fresh sap, which has been absorbed by the roots. The growing point of each bud advances and lengthens out in the direction of its own axis, and thus gives birth to a young twig; this, as it pushes forwards, forms leaves and buds, just in the same manner as we have described to take place on the parent stem during the previous year.* Each twig or branch is in every respect a type and repetition, as it were, of the stem from which it grows. Their structure is the same; their manner of growth is the same; and their mode of propagation is the same. Hence it has been very happily remarked that "plants develope themselves out of themselves progressively."

While the buds are sending out their shoots, and while these are covering themselves with fresh leaves and with embryo buds in the axils of the leaves, the deposition of ligneous matter is constantly going on, from the bases of the leaves and buds, between the bark and the wood of the preceding year. In this manner the second ring of wood is formed. It is of importance that this process of the deposition of woody matter be well understood, as many errors have been committed on this subject by vegetable physiologists at different times. It is now almost universally admitted that the young wood is formed and deposited from the bases of the leaves, by the sap returning along their petioles, after it has been exposed in their blades to the vivifying action of the air. From this account it follows, that *cæteris paribus*, in proportion as the foliage of a plant is abundant, so also will be the formation of new wood; and that the number of strata or annual circular deposits in a branch must always be greater at the lower (nearest the trunk) part of an old branch, than its upper or growing extremity—because, as explained above, there has been a new layer or deposit each successive year, at the period of foliation in the Spring months.

---

* It thus appears that each bud may be considered as a distinct and independent point of formation, and that it is in fact the rudiment of a shoot, which will be developed in the succeeding year. Hence the more buds which are formed on a plant one year, the more abundant will be the growth of branches next year.

Having thus briefly considered the developement of the leaves, buds, and branches, it now remains to explain the very curious and interesting subject of the inflorescence or production of the flowers of a tree. The following description from Lindley is so excellent, that we shall present it in the author's own words:

"After a certain number of years the tree arrives at the age of puberty: the period at which this occurs is very uncertain, depending in some measure upon adventitious circumstances, but more upon the idiosyncrasy, or peculiar constitution of the individual. About the time when this alteration of habit is induced, by the influence of which the sap or blood of the plant is to be partially directed from its former courses into channels in which its force is to be applied to the production of new individuals rather than to the extension of itself;—about this time it will be remarked that certain of the young branches do not lengthen, as had been heretofore the wont of others, but assume a short stunted appearance, probably not growing two inches in the time which had been previously sufficient to produce twenty inches of increase. Of these little stunted branches, called *spurs*, the terminal bud acquires a swollen appearance, and at length, instead of giving birth to new leaves, produces from its bosom a cluster of flower-buds, or *alabastri*, which had been enwrapped and protected from injury during the previous winter by several layers of imperfect leaves, now brought forth as bracts. Sap is impelled into the calyx through the pedicle by gentle degrees, is taken up by it, and exposed by the surface of its tube and segments to air and light; but, having very imperfect means of returning, all that cannot be consumed by the calyx is forced onwards into the circulation of the petals, stamens, and pistil. The petals unfold themselves of a dazzling white tinged with pink, and expose the stamens; at the same time the disk changes into a saccharine substance, which nourishes the stamens and pistil, and gives them energy to perform their functions." 241.

The impregnation of the germen by the pollen of the anthers then takes place; and, when this has been completed, the petals—whose primary use is to enclose and defend the tender organs within—and stamens fade and gradually fall off. All the sap, therefore, which had been directed towards the flower is now expended upon the young fruit, until it attains perfect maturity. Then a new series of changes begins. The sap, which in the Autumn is much less abundant, and more inert in its current, than it had been during the Summer and Spring months, ceases to flow through the peduncle to the ripe fruit. The consequence of this change is, that the peduncle or stalk withers, and at length breaks across either from the weight of the fruit, or from the agitation of the wind; and thus the seeds, enveloped in their moist and fleshy coverings, are committed to the earth, there to remain, until quickened to germination on the approach of the following Spring.

Such is a brief account of the series of phenomena to be observed in the developement of the higher tribes of plants, from the germination of the seed to the period when they form seed of their own, for the propagation of the species.

The most incurious observer cannot fail to admire the beautifully simple means which Nature appears to employ to effect these wonderful changes; and thus the mind is gently, but most irresistibly led to the animating conclusion, that all is formed, and sustained, and regulated by One great Mind, of Almighty Wisdom and of all-providing beneficence.

We are now prepared better to understand the discoveries of vegetable Morphology, or, to use the words of Dr. Lindley,

"That part of botany which treats of the gradual transmutation of leaves into the various organs of a plant, which shows that bracts are leaves affected by the vicinity of the fructification, that the calyx and corolla are formed by the adhesion and verticillation of leaves, that the filament is a form of petiole, and the anther of blade; and, finally, that the ovary itself is a convolute leaf, with its midrib lengthened into a style, and the extremity of its vascular system denuded under the form of stigma." 524.

To Linnæus belongs the merit of having first clearly announced and illustrated this most curious subject. Dr. Lindley has with great propriety given numerous extracts from the writings of the immortal Suede, in which the metamorphosis of the organs of plants is treated of; and we therefore cannot do better than select a few of these for the gratification of our readers. In the Systema Naturæ we find the following expressions:

"Leaves are the creation of the *present* year, bracts of the *second*, calyx of the *third*, petals of the *fourth*, stamens of the *fifth*, and the stamens are succeeded by the pistil." 524.

"Bracts are nothing but leaves which would have been developed another year, if the plant had not flowered." 526.

"The leaves of the calyx are often very small, juiceless, and different from those of the stem, as if scales of buds previous to their developement; but that they still are nothing but leaves of the same nature as those of the stem, must be concluded from this, that when plants, roses, and Geum rivale for example, become, in consequence of excessive nutriment, proliferous, the calycine leaves, which before were small and dry, expand into leaves in size, colour, figure, texture, and substance, exactly like those of the stem. Hence it is not to be doubted, that the calyx and the leaves of the stem were in the beginning alike." 527.

"As the calyx is nothing but leaves, and as each leaf contains in its axil the rudiment of a plant consisting of the rudiments of leaves of a future year, it follows that the petals are of necessity the rudiment immediately within the calycine leaves; the petals, therefore, would have been leaves another year, if flowers had not been produced." 527.

"From double flowers it is apparent that stamens do change into petals and petals into calyx. This is so well known that it need not be insisted on. Now, as from the axil of every leaf arises the rudiment of a plant, and from the axil of the calyx are produced the petals, which are nothing but more tender leaves, and as these petals must have, like other leaves, the rudiments of leaves in their axils, it follows that stamens are so, for they can be transmuted into petals, as the petals can into the leaves of the calyx." 527.

It is uncertain whether Linnæus had satisfied himself of the analogy of the pistil, in point of structure and origin, with the other appendages of the plant. It is, however, most clearly evident from the above extracts, what an enlarged view he had of what may be justly called the philosophic anatomy and physiology of the vegetable kingdom; and we cannot but be surprised at the total neglect into which this most interesting theme of inquiry fell after his death, until Goethe, whose mighty mind had subjected almost every department of scientific and literary research to its sway, re-awakened the attention of botanists, 40 years afterwards, by his celebrated Essay on the Metamorphosis of Plants (Versuch die Metamorphosen der Pflanzen Zu erklären, 1790).* The subsequent researches of De Candolle,

* The readers of our last number know that to Goethe we owe the discovery of the existence of an inter-maxillary bone in the early human fœtus.

Brown, and Petit Thouars, have beautifully confirmed and illustrated the most important of its conclusions, and have established, beyond reach of cavil, that all the appendages of a plant—the scales, leaves, bracts, calyx, corolla, stamens, pistil and fruit—are only modifications of one structure or organ. The type of this primary structure is the leaf, as very clearly appears from the circumstance that all the other parts have a tendency to become leaves, under certain conditions of growth and cultivation.

We shall now endeavour, very briefly, to prove the truth of this proposition, by a few illustrative remarks, commencing with the *Scales*, or those appendages which, arranged in an imbricated manner, cover and protect the leaf-buds of most trees of cold climates. These scales vary much in appearance in different plants. Thus in the *Beech* they are thin, smooth, and dry; in many *Willows* they are covered with a thick down; and in the well known *Populus balsamifera* they exude a viscid tenacious juice. But however much they vary in appearance and outward characters, their proper nature is invariably the same in all plants. They are, in truth, merely abortive or rudimentary leaves; produced at the close of the season, when the flow of the sap is already tardy, and the vital powers of the plant are feeble and inactive. That such is their true nature is obvious from the gradual transition or metamorphosis from scales to perfect leaves observed in different species of the Viburnum, Æsculus, Magnolia, &c. We refer our readers to Dr. Lindley's work for a more minute description of this change, and proceed to the consideration of the next appendage, the *Stipules*. These are processes which, it is well known, are situated at the base of each leaf-stalk or petiole; sometimes investing it like a sheath, at other times being separate and detached from it.

In pinnated leaves we often find stipules at the base of each leaflet, in addition to the larger one belonging to the common petiole. In reference to their true nature, Dr. Lindley says:

"I am clearly of opinion that, notwithstanding the difference in their appearance, they are really accessory leaves; first, because occasionally they are transformed into leaves, as in Rosa bracteata, in which I have seen them converted into pinnated leaves; secondly, because they are often undistinguishable from leaves, of which they obviously perform all the functions, as in the Lathyrus, Lotus, and many other Leguminosæ: and, finally, because there are cases in which buds develope in their axils, as in Salix, a property peculiar to leaves and their modifications." 121.

The *Bracts* or *Bracteæ*, the next part to be considered, are those (usually) leaf-like appendages, which are situated between the proper-leaves and the flowering bud of a plant. They are very generally smaller than the leaves, and also differ from them in their outline, colour, &c. In some plants they are so like to the segments of the calyx, that it is not easy to distinguish between the two. This resemblance is very great in the Camelia. In umbelliferous and compound plants, the bracts and bractlets obtain the names of involucra and involucella. The cup of the acorn is a bract or involucre; so also are the coriacious envelope of the hazel nut, the scales of catkins, the spathæ of palms, &c. and the glumes of grasses.

We see, therefore, that the appendage, to which the general name of *bract* has been given, assumes a multitude of different forms and characters; but in spite of this, all the best botanists of the present day regard them as mere modifications of the proper leaves. That such is their true nature is apparent from their assuming in some plants, as in many of the varieties of the rose, the exact appearance of the leaves. It has been thought by some that bracts may be distinguished from proper leaves by their not producing buds in their axils; but Dr. Lindley has clearly shewn the inaccuracy of this objection, by reference to a multitude of examples in which genuine buds are formed in the axils of bracts, as well as of leaves.

We come now to the consideration of the organs of Inflorescence or Fructification—the calyx, corolla, stamen, pistil and germen—and we shall endeavour to shew that these parts also are not to be regarded as formations essentially distinct and independent from each other, but are rather mere modifications of the leaf, in the same manner as the scales of buds, and the stipules and bracts have been proved to be so. We have already stated that when flower buds are formed at the growing extremity of a twig, the growth or extension of that twig ceases; in consequence, it would appear, of the sap being now altogether directed to the development of the future fruit, and of the energies of the plant being diverted "from increasing the individual to multiplying the species." We are therefore almost *à priori* led to expect an analogy between the parts of a flower bud and those of a leaf-bud; and this conjecture receives confirmation from the well known fact that when a plant, which has perhaps flowered and fruited for several successive years, is transferred to a richer soil and warmer exposure, it often breaks forth into branches instead of producing flowers. Hence arose the remark of Linnæus, "that it appears that branches and leaves can be produced from the provision made for flowers, provided circumstances are favorable to their development."

With respect to the *calyx*, we might adduce a host of examples to shew that its segments, or sepals, often assume all the characters of the proper leaves of the plant. This is especially the case with what are called double or montrous flowers, in which the stamens and pistils have become converted into petals.

What is true of the calyx is equally so of the *corolla*. Botanists used to trouble themselves with making an arbitrary distinction between these two parts; and numerous have been the attempts to draw the line of demarcation between them by definition.

All such attempts are now admitted to be quite nugatory; and we cease to wonder at this, when we remember that the calyx and the corolla are strictly, and in every respect, analogous to each other in origin and in formation; both being mere modifications of the proper leaf. In many plants it is impossible to distinguish between them; they are alike in form, colour, texture and odour. That the petals of the corolla are occasionally converted into leaves is admitted by all botanists. We cannot afford room to adduce individual examples, and shall therefore haste on to the consideration of the essential parts of fructification—the *stamens* and *pistils*.

That the former are, in point of structure and formation, merely modifications of the petals of the corolla, may be distinctly and very easily traced in the number of plants, especially when double flowers are produced. The following extract from Lindley is very interesting.

"In double roses the precise nature of this metamorphosis is shown in a very instructive way: if any double rose is examined, it will be seen that those petals which are next the stamens contract their claw into the form of a filament; a distortion of the upper part, or limb, also takes place; the two sides become membranous, and put on the colour and texture of the anther; and sometimes the perfect lobe of an anther will be found on one side of a petal, and the half-formed mis-shapen rudiment of another on the opposite side." 536.

The transformation of stamens into petals is still more conspicuous in double flowers of the common Columbine of our gardens. In some rare cases the stamens have been found converted into green leaves. Roper witnessed this singular phenomena in a variety of the Campanula, and Petit Thouars, in the Brassica Napus.

With respect to the *pistil*, (under which term we include the germen or ovary, as well as the style and stigma,) nothing is more common than to find it converted into genuine petals; as, for example, in double Narcissi, Wall-flowers, Saxifrages, &c. In some plants, the germen is seen to resemble very closely the segments of the calyx; and, in others, a miniature leaf, as in many roses, and more conspicuously in the common double cherry. The exact analogy between the structure of the pistil and that of the proper leaves may be very beautifully traced in the gradual development of the former part in leguminous plants. Take the pea, for example, and examine its pistil. The young pod is seen to consist of its two laminæ, which are joined together by sutures along their longitudinal edges, and from whose united extremity proceeds the tapering style, terminated by the stigma. The seeds are found to be attached within to one only of the sutures of the pod, and the style is observed to be prolonged or continued out from the other. If we slit the young pod open along the line of the former structure, and then compare it with a leaf of the plant, we shall at once perceive a marked resemblance between these two parts—the seed-bearing suture corresponding to the edges of the leaf, the other suture to its midrib, and the style to its mucro or point.

We must here close this interesting train of enquiry; and we cannot do it better than by giving a short extract from the celebrated Essay of Goethe, to whom philosophical botany is so deeply indebted.

"Keeping in view the observations that have now been made, there will be no difficulty in discovering the leaf in the seed-vessel, notwithstanding the variable structure of that part, and its peculiar combinations. Thus, the pod is a leaf which is folded up, and grown together at its edges, and the capsule consists of several leaves grown together; and the compound fruit is composed of several leaves united round a common centre, their sides being opened so as to form a communication between them, and their edges adhering together. This is obvious from capsules, which, when ripe, split asunder; at which time each portion is a separate pod." 540.

It may be expected, before closing this brief sketch, that we should say something of the works, which appear at the head of the present article.

By the free use, which we have made of Dr. Lindley's Introduction, our readers will have already decided what is our opinion of its merits. It is indeed a work of classical excellence; most accurate, instructive, and pleasing. It is essential for every botanist to have a copy of it beside him. We wish that we could speak quite as highly of M. Raspail's System. It abounds certainly with much ingenious speculation, and contains at the same time a large mass of interesting details. For these reasons it well deserves the patient perusal of the more advanced students of botany. The following abstract of its contents will shew our readers, what they may expect from its perusal.

The first part is occupied with a description of vegetable nomenclature, and extends to upwards of 100 pages. The second part is devoted to a very minute investigation of what is termed Organogeny, or the development of vegetable organisation. Had our limits permitted, we should have drawn largely from this section of the work, as the author treats very ably, although rather in too speculative and dogmatic a tone, of the successive steps or processes, by which the evolution of the various parts of a plant is accomplished. Many of his remarks are very ingenious, and the observations and experiments, which he relates, deserve the utmost attention from the philosophical botanist.

The second volume commences with the physiology of vegetable life. The influences of light, water, air, atmospheric changes, soil, manure, &c., not only upon the vegetation of plants in general, but upon each particular organ of a plant, are detailed with great industry. The fourth part considers the various systems of classification, natural and artificial, which have been proposed by different authors; and the fifth and last part explains the practical applications of a correct knowledge of vegetable physiology, not only to the successful culture and propagation of plants, but to various departments of other arts and sciences.

Our readers will thus be enabled to judge for themselves in some respects of this new work from M. Raspail. He is very ingenious in all his novelties; and even his errors announce him to be a man of genius. The accompanying volume of plates deserves the very highest praise; the drawings and engravings are quite admirable. The work is published by M. Bailliere, who seems to be quite the Coryphæus of medical booksellers, and to whom we are generally indebted for all the most useful publications from the Parisian press.

